# Phenotypic and transcriptomic responses of diverse rice accessions to transient heat stress during early grain development

**DOI:** 10.3389/fpls.2024.1429697

**Published:** 2024-08-15

**Authors:** Anil Kumar Nalini Chandran, Puneet Paul, Balpreet K. Dhatt, Jaspreet Sandhu, Larissa Irvin, Shohei Oguro, Yu Shi, Chi Zhang, Harkamal Walia

**Affiliations:** ^1^ Department of Agronomy and Horticulture, University of Nebraska, Lincoln, NE, United States; ^2^ Department of Biological Science, University of Nebraska, Lincoln, NE, United States; ^3^ Center for Plant Science Innovation, University of Nebraska, Lincoln, NE, United States

**Keywords:** *Oryza sativa*, heat stress, transcriptome, grain development, unfolded protein response

## Abstract

Heat stress (HS) occurring during the grain-filling period has a detrimental effect on grain yield and quality in rice (*Oryza sativa*). The development of heat-resilient cultivars could partly solve this issue if tolerant alleles can be identified and incorporated into the germplasm. In this study, we posit that some of the phenotypic variations for heat resilience during grain development could be due to variations in gene expression among accessions. To test this, we characterized the HS response of 10 diverse rice accessions from three major sub-populations using physiological and transcriptome analyses. At a single-grain level, grain width and grain thickness emerged as the most heat-sensitive traits. During a transient HS, IND-3 was categorized as highly sensitive, while five accessions exhibited moderate heat sensitivity, and four accessions were tolerant. Only a core set of 29.4% of the differentially expressed genes was common to the three rice sub-populations. Heat-tolerant accession TEJ-5 uniquely triggered an unfolded protein response (UPR) under HS, as evident from the induction of *OsbZIP50* and downstream UPR genes. *OsbZIP58*, a gene that positively regulates grain filling, was more highly induced by HS in IND-2 despite its moderate heat sensitivity. Collectively, our analysis suggests that both unique gene expression responses and variation in the level of responses for a given pathway distinguish diverse accessions. Only some of these responses are associated with single-grain phenotypes in a manner consistent with the known roles of these genes and pathways.

## Introduction

Climate change-driven rise in global surface mean temperature and frequent heat waves are predicted to compromise crop production and food security. The mean global surface temperature is projected to increase by up to 4.8°C by 2100 (Song et al., 2022). In addition to the rise in temperatures, shrinking farmland and exponential population growth are further exacerbating this situation. An estimated 70% increase in crop production is needed to meet global food consumption by 2050 (Delaney, 2015). The impact of heat stress (HS) on reproductive development is particularly severe and is a significant contributing factor to food insecurity, as a disproportionate level of food consumption depends on reproductive organs such as grains. Even transient episodes of HS during reproductive development can substantially reduce yield and grain quality in major staple crops (Prasad et al., 2017; Dhatt et al., 2019; Jagadish, 2020; Schittenhelm et al., 2020; Paul et al., 2020b; Niu et al., 2021). A yield reduction of 6% in wheat, 3.2% in rice, 7.4% in maize, and 3.1% in soybean is estimated for every 1°C rise in temperature (Zhao et al., 2017). Rice (*Oryza sativa*), a staple source of nutrition for nearly 3.5 billion people, is particularly prone to HS, as some of the fastest warming trends have been observed in Southeast Asia, a major rice-growing region (Muthayya et al., 2014; Wing et al., 2018). For instance, nearly 24 million hectares of irrigated rice farms in Asia, accounting for 40% of the global rice supply, experience a monthly average daytime temperature above 33°C during the reproductive stages (Welch et al., 2010).

Endosperm constitutes the major source of nutrition in most grains, as it serves as the sink tissue for the influx of photoassimilates from source organs. Carbon partitioning, accumulation rate, and duration of storage compounds at the early grain-filling stage influence the mature grain size and weight (Garg et al., 2017). Post-fertilization, a series of grain developmental events are initiated, involving rapid caryopsis expansion during the coenocytic stage, during which rapid mitotic division of triploid endosperm nuclei occurs without the formation of cells (Wu et al., 2016). This is followed by cellularization around the periphery of the central vacuole and simultaneous cytokinesis and cell wall formation, as the entire vacuolar space is filled with cellularized cells. These cells then accumulate grain storage compounds such as starch, proteins, and lipids during the grain filling (Wu et al., 2016). The heat sensitivity of rice developmental stages is evident from the observed precocious cellularization under moderate HS (35°C/30°C) and delayed cellularization when grains are developing under severe HS (39°C/35°C) (Folsom et al., 2014). The heat sensitivity of developing grains increases at phenotypic levels when HS occurs during early grain-filling stages (Sandhu et al., 2021). In rice, the highly heat-sensitive coenocytic and cellularization stages occur within 24–96 h after fertilization (HAF). Perturbing the timely transition from the coenocytic to cellularization phase impairs grain development to varying degrees. Effects of the impaired grain-filling process are manifested in the form of grain abortion, reduction in grain size, or grain chalkiness coupled with distorted packaging and altered levels of storage compounds. Therefore, elucidation of the genetic determinants of early grain heat sensitivity is a critical component of increasing our understanding of the basis of HS response variation in rice.

Genes involved in grain developmental processes have been previously investigated in rice under ambient and HS conditions (Liu et al., 2010; Liao et al., 2015; Sreenivasulu et al., 2015; Sun et al., 2015; Wang et al., 2019; Dhatt et al., 2021; Ren et al., 2022). A reduction in grain size, weight, and quality under HS has been shown to be caused by the misregulation of genes related to phytohormones, sugar transport, starch and protein metabolism, and several grain-filling transcription factors (Hara et al., 2015; Huang et al., 2017; Wu et al., 2019; Tabassum et al., 2020; Ren et al., 2021; Cao et al., 2022; Ravikiran et al., 2022). Polycomb Repressive Complex 2 (PRC2) member *Fertilization-Independent Endosperm1* (*OsFIE1*) is a central syncytial stage regulator that negatively affects grain size increase under HS (Folsom et al., 2014). Allelic variation at the *OsFIE1* locus has been shown to regulate natural variation in high-temperature response in rice germplasm (Dhatt et al., 2021). Type 1 MADS-box genes such as *OsMADS78*, *OsMDAS79*, and *OsMADS89* control grain size and shape and are regulated in a PRC2-dependent manner. *OsMADS89* facilitates the nuclear localization of *OsMADS78* and *OsMADS79*, which are involved in regulating the rate of endosperm cellularization (Paul et al., 2020a). Although phenotypic diversity has been characterized for rice reproductive development under HS, the extent of molecular variation among diverse genotypes remains unexplored (Dhatt et al., 2021; Chandran et al., 2022). An insight into the transcriptomic response of accessions with different HS tolerance levels facilitates the mining of favorable alleles as targets for breeding HS-resilient cultivars. A comparative analysis of transcriptomic signatures of diverse germplasm may also be used to understand the genetic basis of HS tolerance; however, a similar study dissecting the transcriptome of early grain-filling stages of diverse accessions under HS is not explored.

Rice diversity panel 1 (RDP1) accessions exhibit significant phenotypic variations in response to HS occurring during the early grain developmental stages (Paul et al., 2020b; Dhatt et al., 2021; Kumar et al., 2021; Chandran et al., 2022; Bi et al., 2023). In this study, we combined precise single-grain-level phenotypic responses to a transient HS and a time course transcriptome analysis of grains during the two heat-sensitive developmental stages, coenocytic and cellularization, for 10 diverse rice accessions. We identified a range of variations in phenotypic and molecular responses across these rice accessions. We found a heat-responsive unfolded protein response pathway to be characteristic of a tolerant accession. We also found that HS triggered an enhanced grain-filling response, unlinked to developmental acceleration in an accession that is not heat-tolerant. Collectively, our analysis highlights diverse genotype-specific molecular pathways triggered by HS, but none of them are strong predictors of either tolerance or sub-population specificity. This suggests that multiple pathways are independently associated with single-grain-level heat resilience under a transient HS.

## Materials and methods

### Plant material and growth conditions

Ten rice accessions were selected from RDP1 as subjects of the study (**Supplementary Table S1**). The accessions were selected based on their relatively similar flowering time and genetic and geographical diversity. The grains were de-husked, sterilized with bleach (40%, v/v), and rinsed with sterile distilled water. Sterilized grains were germinated in the dark on half-strength Murashige and Skoog media. After germination, 1-week-old seedlings were transplanted in Greenhouse 1 into 4-inch square pots with soil until flowering. Greenhouse 1 conditions were set to 16-h light and 8-h dark at 28°C ± 1°C and 23°C ± 1°C, respectively, with 55%–60% humidity throughout the growth period.

### Heat stress treatments

At the beginning of fertilization, the florets were marked to ensure precise tracking of developmental progression. Plants with marked florets were divided into the control and HS groups. The HS group was divided into three sub-groups to set up a time series transient HS treatment. In the transient HS treatment, at 1 day after fertilization, all stress sub-group plants were moved to Greenhouse 2 and exposed to high day and night temperature treatment (HDNT; 16-h light and 8-h dark at 36°C ± 1°C and 32°C ± 1°C, respectively). The three sub-groups were treated for 48 h, 72 h, or 96 h of HS and returned to Greenhouse 1 to grow until maturity. Control plants remained in Greenhouse 1 until maturity.

### Grain morphometric analysis

The grains were harvested at maturity approximately 4–5 weeks after marking the spikelets. Harvested grains were dried in an oven (28°C) for 2 weeks, and dehusked grains (Automatic Rice Husker TR-250) were collected for imaging. The dehusked grains were scanned using an Epson Expression 12000 XL scanner (Epson America Inc., Los Alamitos, CA, USA) at 600-dpi resolution. Scanned images were processed using a MATLAB application, SeedExtractor (Zhu et al., 2021). For each accession, marked spikelets from 10–21 plants were used per treatment (**Supplementary Table S1**).

### Histochemical analysis

Developing grains (72 HAF and 96 HAF) from the control and HS groups were collected and fixed in 1 mL of FAE solution [formaldehyde (2%), acetic acid (5%), and ethanol (60%)] for 16 h. After being washed with 70% ethanol, samples were stored overnight at 4°C. This step was followed by a dehydration series with ethanol (85%, 95%, and 100%) for an interval of 1 h each at room temperature, followed by xylene (100%) for 2 h. Samples were incubated in 500 µL of a 1:1 mixture of xylene and paraplast tablets overnight at 60°C (Paul et al., 2020b). Subsequently, samples were embedded in the paraplast. Microtome sections (10 µm) from paraplast molds with embedded samples were obtained using a rotary microtome (Leica RM2125 RTS). Section fixed on slides were dewaxed and stained with toluidine blue. The processed slides were observed using a bright-field microscope (Leica DM-2500). A minimum of three sample replicates from two independent plants were used for this assay.

### Sample collections and RNA extraction

The developing grains at 48, 72, and 96 HAF time points were collected from control and HS treatments for 10 accessions using sterile forceps. The samples were flash-frozen using liquid nitrogen. The samples were homogenized (TissueLyser II, Qiagen, Valencia, CA, USA), and total RNA was extracted using the RNeasy Plant mini kit (Qiagen) following the manufacturer’s instructions.

### RNA-seq analysis

A total of 120 RNA-Seq libraries that constituted approximately 6.4 billion reads were generated using the Illumina sequencing platform (**Supplementary Table S2**). RNA samples were processed, and libraries were prepared with TruSeq Stranded mRNAseq sample preparation kit (Illumina, San Diego, CA, USA). Libraries were quantitated using qPCR and one lane for 151 cycles from one end of the fragments on a NovaSeq 6000 using a NovaSeq S4 reagent kit. Illumina bcl2fastq software was used for fastq file conversion and sample demultiplexing. The resultant 150-nucleotide single-end reads were trimmed using Trimmomatic (Bolger et al., 2014) and mapped to the rice reference genome (RGAP v7.0) using HISAT2 aligner (Kim et al., 2019). The average mapping rate was approximately 92%. Reads mapped to multiple locations were omitted, and the number of reads that mapped to the exon regions was estimated using the featureCounts read summarization program (Liao et al., 2014). Reads were normalized, and genes with consistently low expression, <5 reads in all samples, were removed. The differentially expressed genes (DEGs) were estimated using the edgeR package (Robinson et al., 2009). A multifactor design was adopted to compare different treatment groups using the “makeContrasts” function.

### Network analysis and module detection

A total of 120 RNA-Seq samples from grain developmental stages were used for constructing a co-expression network using the WGCNA R package (Langfelder and Horvath, 2008). In total, 29,943 genes with variance stabilizing transformation were used for module detection. A soft power threshold of 12 was chosen to transform the correlation matrix into a signed weighted adjacency matrix using the blockwiseModules function with additional parameters, minModuleSize = 30, mergeCutHeight = 0.25. The resulting adjacency matrix was then used to calculate the topological overlap matrix. The network was exported using the function exportNetworkToCytoscape and visualized using Cytoscape version 3.9.1.

### Statistical analysis

Principal component analysis for transcriptome data was conducted using the ggfortify package in R software version 3.6.2 (R Core Team 2021). Significance levels for grain morphometrics between the control and HS groups were estimated using the t.test function in R 3.6.2.

## Results

### Grain width and thickness are highly sensitive to transient heat stress

**Figure 1:**
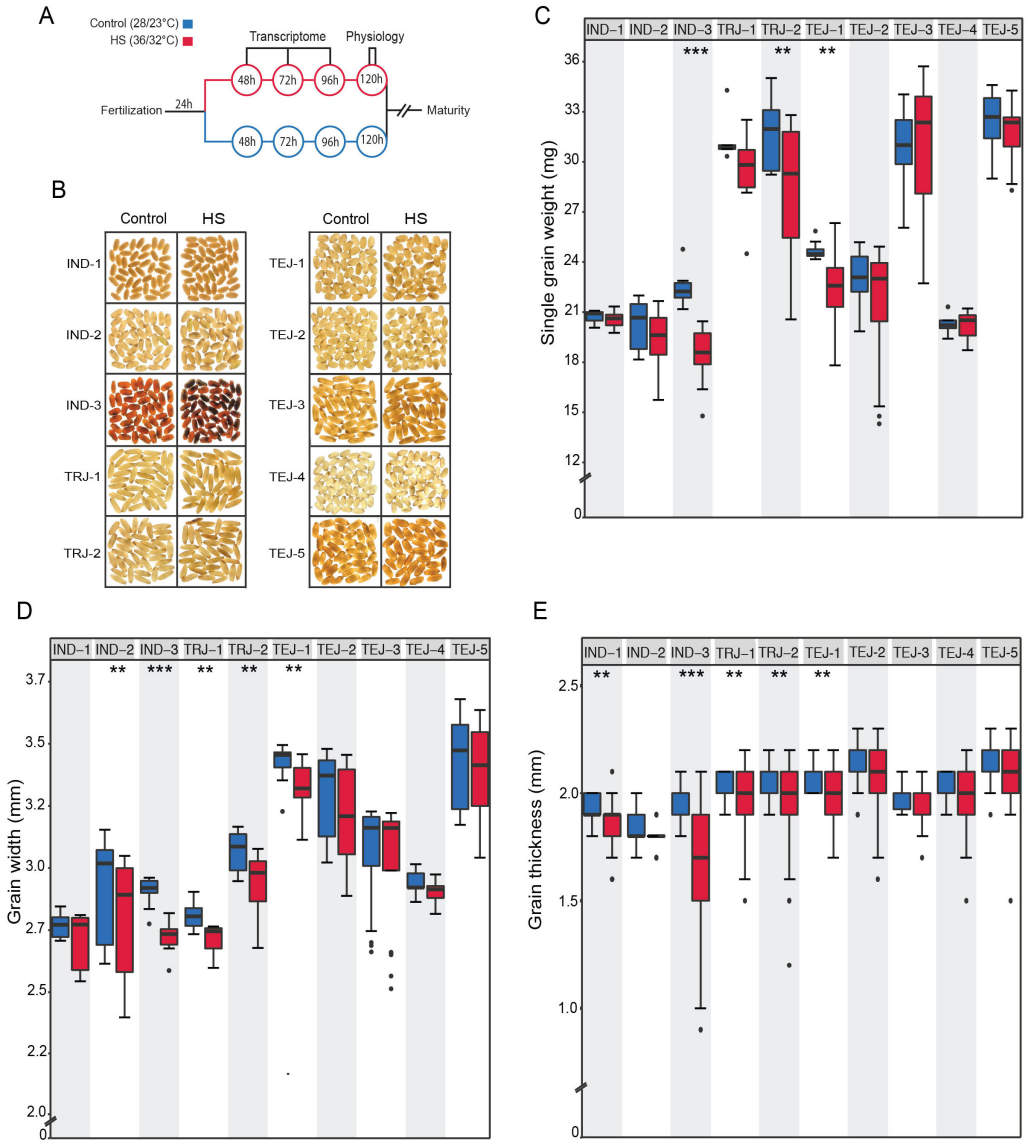
Transient heat stress (HS) at the early grain-filling stage exhibited significant mature grain phenotypic differences among 10 diversity accessions. **(A)** Schematic diagram depicting the experimental setup. **(B)** Representative mature grain images from control and heat‐stressed accessions. Plants marked at the time of fertilization were imposed HS (36°C/32°C) after 24 h for a period of 96 h and moved back to control (28°C/23°C) until maturity or plants remained in control throughout the grain development. **(C)** Grain morphometric analysis showing the HS sensitivity of accessions to single-grain weight. **(D)** Grain width. **(E)** Grain thickness. *N* = 10–21 plants per treatment for each accession. The statistical significance of the differences in grain size for the two treatment groups was estimated using paired *t*-test (***p < 0.0001 and **p < 0.05).

We evaluated the phenotypic responses to a transient HS in 10 accessions at the early grain-filling stage by exposing them to HS for 96 h, beginning at 24 HAF and ending at 120 HAF (**Figures 1A, B**). We performed precise tracking of the grain developmental stage by marking individual grains from HS-treated and control plants at the onset of fertilization. We define “tolerance” and “sensitivity” to HS in the context of single-grain size phenotypes for this study. We analyzed HS-treated mature grains along with corresponding controls. We used grain image analysis to obtain mature grain size features. This analysis showed a genotypic diversity in response to transient HS (**Figure 1**). HS caused a significant reduction in single-grain weight (sgw) in IND-3, TRJ-2, and TEJ-1 (**Figure 1C**). It is noteworthy that grain length is largely unaffected by HS among accessions except for a significant reduction in IND-3 (*p* < 0.001) (**Supplementary Figure S1A**). In contrast, HS negatively impacted the grain width of five accessions (IND-2, IND-3, TRJ-1, TRJ-2, and TEJ-1; **Figure 1D**). Similar to grain width, our analysis also showed that IND-1, IND-3, TRJ-1, TRJ-2, and TEJ-1 significantly reduce grain thickness in response to HS (**Figure 1E**). These results indicate that grain width and thickness are more sensitive to HS than grain length during the early grain development window. It is notable that while IND-3 showed high sensitivity in terms of a significant reduction in all measured grain properties under HS, IND-1 and IND-2 showed moderate sensitivity to HS. Although we used a relatively small set of accessions for this study, these observations suggest that the IND sub-population may have considerable phenotypic variations in response to HS. None of the selected accessions showed a significant difference in grain fertility under HS for the marked grains. This could be due to our strategy of waiting for 24 HAF to initiate the HS. Our rationale for this approach is to focus on post-zygotic HS responses in young grains. Based on our analysis, IND-3 is categorized as highly sensitive to HS, whereas IND-1, IND-2, TRJ-1, TRJ-2, and TEJ-1 showed moderate sensitivity to HS. TEJ-2, TEJ-3, TEJ-4, and TEJ-5 exhibited HS tolerance.

Given the differential sensitivity to multiple grain-level parameters, we next examined the impact of HS on grain quality. Grain chalkiness, an important quality trait, is caused by air space among loosely/abnormally packaged starch granules. Chalkiness results in opaque white areas in an otherwise translucent endosperm (Armstrong et al., 2019). We used the red and green pixel ratio (RG) of grains extracted from SeedExtractor to evaluate the grain quality, as RG was demonstrated as an indicator of grain chalkiness (Chandran et al., 2022). The 10 accessions differed in grain chalkiness (**Figure 1B**; **Supplementary Figure S1B**). We found that HS caused severe grain chalkiness in IND-3, TRJ-1, and TEJ-5. Overall, morphometric analysis indicates that the selected rice accessions represent significant phenotypic divergence in HS response for further examination of their underlying molecular responses.

### Sub-populations and heat stress are the primary drivers of early grain transcriptomes

**Figure 2:**
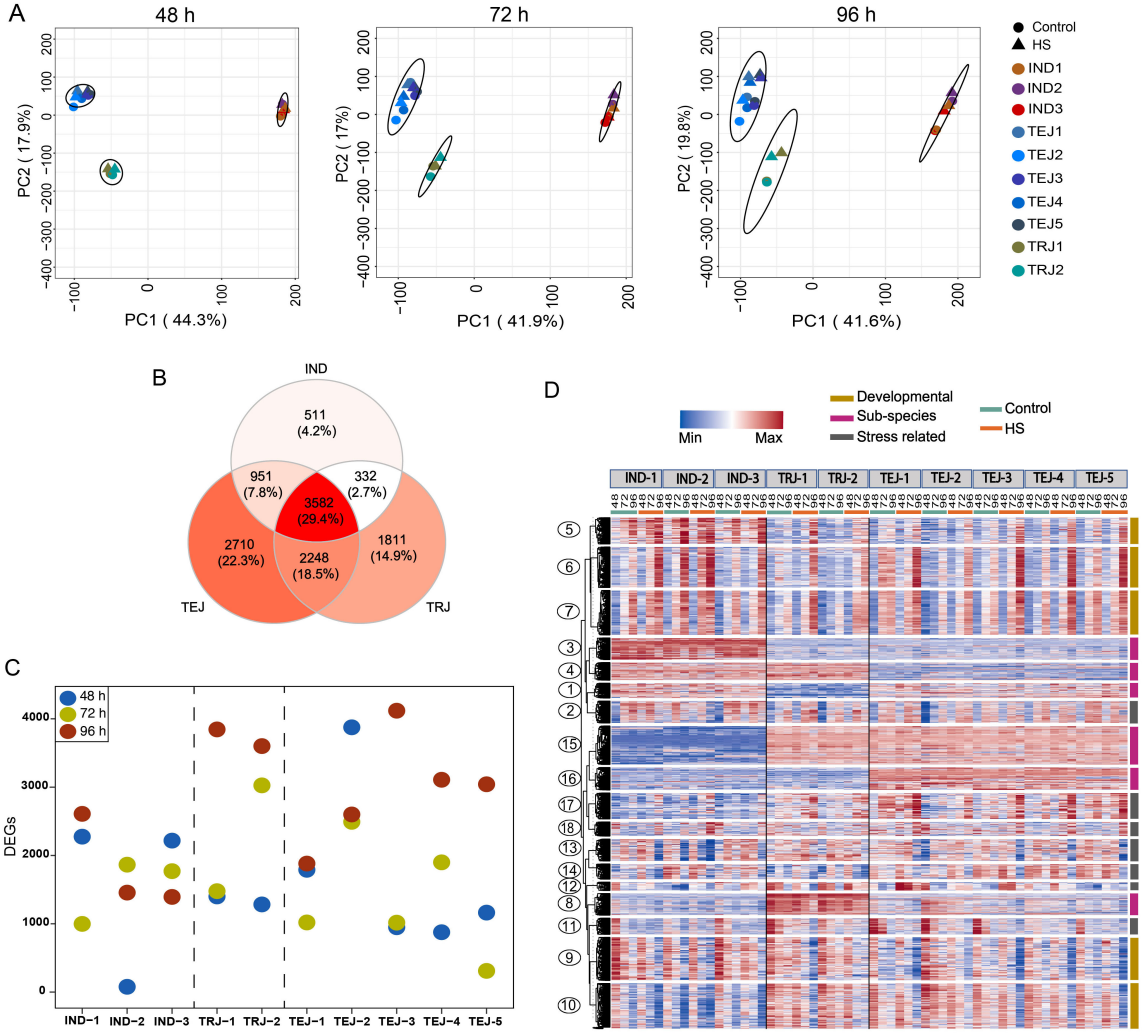
Transcriptome analysis at early grain-filling stages under control and heat stress (HS) revealed the genetic diversity of 10 accessions in grain development and response to HS. The RNA-Seq dataset was generated from developing grains at 48, 72, and 96 h after fertilization (HAF) under control and HS. **(A)** Principal component analysis of the RNA-Seq dataset generated from developing grains at 48, 72, and 96 HAF under control and HS showed that samples are primarily separated based on the sub-population of origin and HS. **(B)** Comparative analysis of differentially expressed genes (DEGs) showed that 3,582 genes are differentially regulated in all sub-populations, whereas 511, 1,811, and 2,710 DEGs were unique to IND, tropical japonica, and temperate japonica, respectively. **(C)** Comparative analysis of the number of DEGs in each accession at each time point. **(D)** Clusters with diverse expression patterns derived from clustering analysis of 12,512 DEGs.

We next examined the transcriptomic responses of developing grains during the period of transient HS by generating RNA-Seq data for the 10 accessions under control and HS treatments at 48, 72, and 96 HAF time points. As evident from morphometric analysis, HS imposed during this early grain development stage has a lasting impact on multiple grain phenotypes at maturity. We performed principal component analysis (PCA) on the normalized gene expression values to determine genotypic relationships and HS-responsive transcriptomic shifts triggered during the developmental progression (**Figure 2A**). We found that PCA1 and PCA2 can cumulatively explain more than 50% of the variation in sample separation under control and HS. In this analysis, IND, TEJ, and TRJ accessions clustered into three distinct groups, indicating that sub-population level differences among the accessions are the primary driver of transcriptional status. The magnitude of HS impact was less for the transcriptome at 48 HAF, as a significant sample separation was not observed in most of the accessions between the control and HS samples. However, the HS responsiveness of the transcriptome was more evident in 72 and 96 HAF samples, as controls and HS-treated samples resolved into distinct sub-clusters. We also observed a difference in sample clustering patterns in response to HS for specific accessions. For instance, TEJ-2 showed HS-dependent sample separation earlier at 48 HAF, whereas IND-2 showed a minimal separation of the treatment groups even at 96 HAF. For all other accessions, 96 h of HS imposed extensive transcriptomic re-programming compared to other time points. However, these transcriptome-level responses at 96 h of HS still do not perturb the sub-population level clusters, which remains the primary driver of clustering.

Next, we estimated DEGs at 48 h, 72 h, and 96 h of HS at the grain developmental stage to identify transcriptome-level differences in stress responses for the 10 accessions. A pairwise comparison of HS-treated samples against their respective controls revealed 6,566, 6,920, and 10,334 DEGs [log_2_FC ≥ 1, false discovery rate (FDR) <0.05] at 48 h, 72 h, and 96 h, respectively. We found a core set of 3,582 genes that were differentially regulated in all sub-populations at least at one time point (**Figure 2B**; **Supplementary Table S3**). We also found 2,710 TEJ-specific, 1,811 TRJ-specific, and 511 indica-specific DEGs (**Supplementary Table S3**). Our DEG estimation also showed the difference in the number of HS-responsive genes among accessions (**Figure 2C**). For instance, we found only 77 DEGs in IND-2 at 48 h, much lower than 3,876 DEGs in TEJ-2 at the same time point. Similarly, we detected only 310 DEGs in TEJ-5 at 72 h compared to 3,026 genes identified in TRJ-2 (**Figure 2C**). In total, we found 12,512 genes that showed differential regulation under HS for at least one time point for at least one accession. We included this gene set for downstream analysis.

Temporal expression patterns of DEGs can provide insights into the phenotypic characteristics observed under control and HS conditions. We aimed to identify genes that are 1) involved in HS tolerance, 2) developmentally regulated in early grain-filling stages, and 3) genes with sub-population-specific patterns. We applied the gap statistics (Tibshirani et al., 2001), which enables the selection of the optimum *k* value in *k*-means clustering, and we performed clustering on 12,512 genes (**Figure 2D**). We analyzed the resultant 18 clusters to address stress-responsive, developmental, and sub-population level variations among the accessions.

### Group A: genes associated with heat stress adaptation

**Figure 3:**
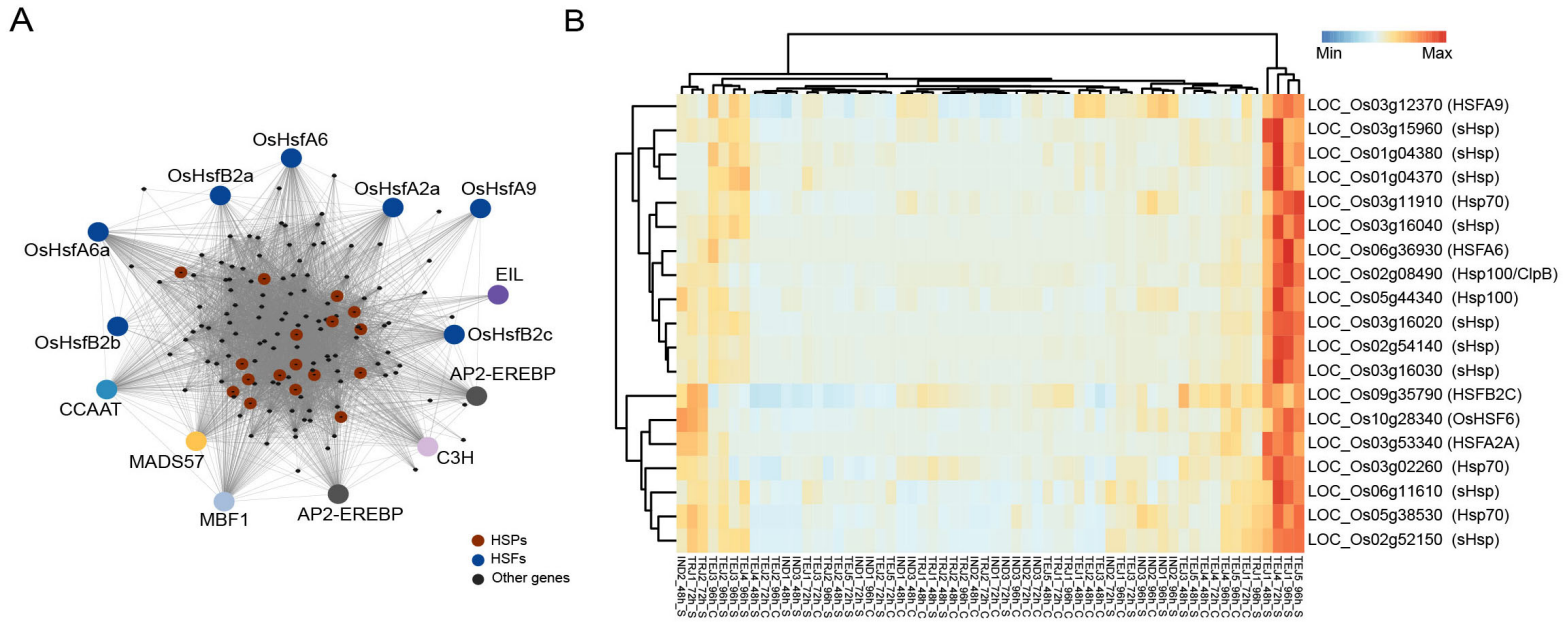
Co-expression analysis of grain-expressed genes revealed a heat stress (HS)-induced pathway mainly constituted by heat shock proteins (HSPs) and associated transcription factors in specific accessions. **(A)** Network of M24 module consisting of heat shock response genes. **(B)** Induction pattern of HSPs and heat shock factors in TEJ-1, TEJ-4, and TEJ-5.

We first examined the gene regulatory pathways activated in response to HS during the grain-filling stage using weighted gene correlation network analysis (WGCNA). Network analysis of 29,943 genes expressed in developing grains yielded 42 co-regulated gene modules with the lowest and highest module sizes of 32 and 8,818, respectively. Among the modules, M24 (144 genes) was enriched with Gene Ontology (GO) terms associated with “protein folding”, “response to HS”, and “heat shock protein binding” (**Figure 3A**), highlighting the significance of this module in HS response (**Supplementary Table S4**). The M24 module consists of 14 TF genes, seven of which are from the heat shock factor (HSF) family. Apart from HSFs, TFs involved in ethylene signaling, such as two APETALA2/ethylene-responsive element binding proteins (*LOC_Os02g34260* and *LOC_Os02g34270*) and an ethylene-insensitive3-like/ethylene-insensitive3 gene (*LOC_Os04g38400*), were members of this network. Ethylene signaling is an integral component of HS tolerance in *Arabidopsis*, where ethylene insensitive 3 (*EIN3*) regulates TFs *ERF95* and *ERF97* to constitute a transcriptional cascade with heat shock family genes in response to HS (Huang et al., 2021a). Although *ERF95* and *ERF97* expressions are not accession-specific, we found that *EIN3* is specifically induced in TEJ-3 at 96 h of HS. The module also consists of 17 of 31 (17/31) heat shock proteins (HSPs) in the rice genome. Since many of the HSFs are co-regulated with HSPs, we asked whether these HSPs–HSFs induce a generic response under HS or whether the response is accession-specific. Our analysis showed that 14 of these HSPs from small HSP, Hsp 70, and Hsp100 families are specifically induced in two tolerant accessions (TEJ-4 and TEJ-5) at 72 h or 96 h of HS and a moderately tolerant accession (TEJ-1) at 48 h and 96 h of HS (**Figure 3B**). In addition to HSPs and HSFs, 91 genes in the module are preferentially expressed in these three TEJ accessions (TEJ-1, TEJ-4, and TEJ-5) in response to HS (**Supplementary Figure S2**).

We then examined the specific regulators of the suite of HSPs and found that *OsHSFA6*, *OsHSFA2A*, and *OsHSF6* (*LOC_Os06g36930*, *LOC_Os03g53340*, and *LOC_Os10g28340*, respectively) are highly co-regulated with HSPs in M24. Notably, a similar HSP–HSF network has also been identified in wheat, where *TaHsfA6f* positively regulates an array of HSPs, leading to thermotolerance at the seedling stage under HS (Xue et al., 2015). These results indicate that an accession-specific tolerance response to HS during early grain-filling triggers a stress response network that involves HSPs–HSFs.

### TEJ-5-specific unfolded protein response in ER

**Figure 4:**
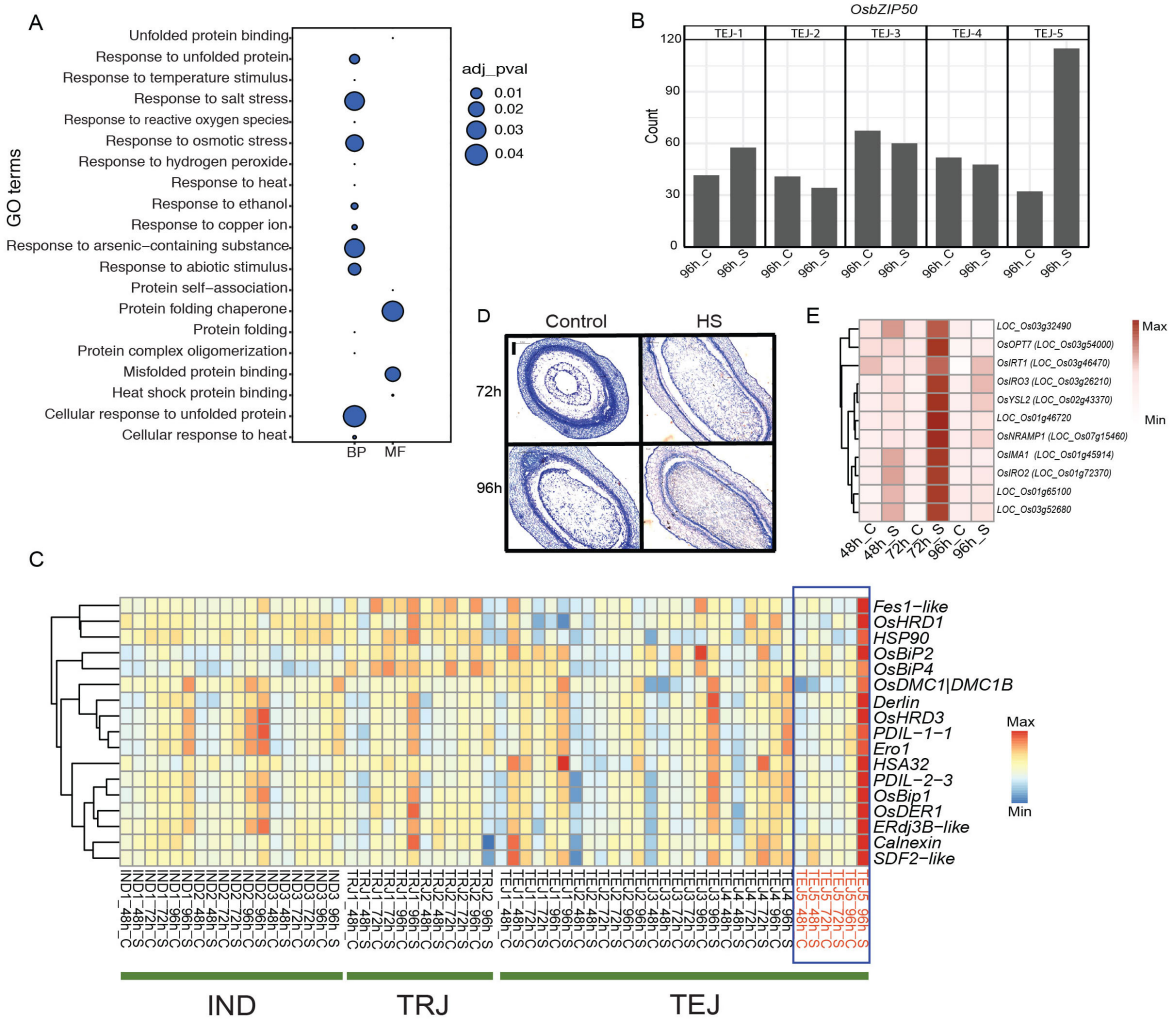
Heat stress (HS) at the grain-filling stage activated unfolded protein response (UPR) in TEJ-5. **(A)** Cluster 17 genes are populated with gene ontology terms related to UPR response. **(B)** HS induced *OsbZIP50* at 96 (h) **(C)** Endoplasmic reticulum resident chaperonin genes are activated by *OsbZIP50*. **(D)** Histochemical analysis shows the accelerated cellularization of TEJ-5 at 72 h of HS. **(E)** Iron homeostasis genes induced in TEJ-5 at 72 h of HS. BP, biological process; MF, molecular function. Scale = 0.2 μm.

Our phenotypic evaluation of 10 accessions identified TEJ-5 as a remarkable accession with HS tolerance and larger grains. We attributed a cluster of genes (cluster 17) enriched in GO terms related to “protein folding”, “endoplasmic reticulum (ER)”, and “response to HS” to TEJ-5. These genes are likely associated with intracellular events for sensing and adapting to HS (**Figure 4A**). Genes in this cluster are preferentially expressed in TEJs at 96 h of HS (**Figure 2D**). However, the expression of the ER stress response gene, *OsbZIP50* (*LOC_Os06g41770*), showed significant induction in TEJ-5 compared to other accessions (**Figure 4B**; **Supplementary Figure S3**). Consistent with its function in rice, orthologs of *OsbZIP50* in maize and *Arabidopsis* (*zmbZIP60* and *AtbZIP60*, respectively) are required for unfolded protein response (UPR) (Li et al., 2012; Takahashi et al., 2012). UPR is generally triggered upon overloading and accumulation of misfolded proteins in the ER, known as ER stress (Oono et al., 2010). Given the HS-dependent induction of *OsbZIP50* in our analysis, we speculate that enhanced ER stress response could be a mechanism to alleviate HS during early grain development. To examine the role of UPR in TEJ-5 stress adaptation, we analyzed the response of key genes in the UPR mechanism that counter HS impact. ER stress sensor *IRE-1* mediates unconventional splicing of *OsbZIP50*, and the spliced form of *OsbZIP50* confers a nuclear localization signal. The activated OsbZIP50 is translocated to the nucleus and regulates multiple UPR genes by binding to the pUPRE-II elements in their promoters (Hayashi et al., 2013). We found that induction of *OsbZIP50* in TEJ-5 resulted in the activation of several downstream ER localized chaperonins (**Figure 4C**). Major UPR pathway chaperonins, including *OsBip1*, *OsPDIL*, and *Calnexin*, have higher expression in TEJ-5 at 96 h of HS. The induction of these chaperonins suggests the occurrence of ER stress and activation of UPR response in TEJ-5. To further elucidate the UPR response, we scanned the 2-kb upstream region of genes with GO terms related to protein folding and HS for the presence of UPR-related regulatory motifs (Ruberti and Brandizzi, 2014). We identified that 74/705 genes harbor the *OsbZIP50*-binding pUPRE-II motif (Hayashi et al., 2013). Additionally, 10 genes contain UPR-related ERSE motifs. The presence of these motifs suggests a higher activation of the UPR network under HS as the basis of heat tolerance in TEJ-5. We scanned the 2-kb upstream and coding region of *OsbZIP50* in 3,000 rice germplasms (Wang et al., 2018) to analyze the allelic variations that may contribute to the expression of *OsbZIP50* (**Supplementary Table S5**). However, we found that the *OsbZIP50* region is highly polymorphic in nature, which limits the possibility of linking allelic variation regulating the expression of *OsbZIP50*.

In the bifurcated cascade for UPR response, ER stress is also perceived by sensors *OsbZIP60* and *OsbZIP39*. Unlike *OsbZIP50*, proteolytic activation of their products regulates several downstream UPR genes. We detected a significant induction of *OsbZIP60* like *OsbZIP50* in TEJ-5 under HS; the expression of *OsbZIP39* did not alter significantly from its basal level in any of the accessions. The relative expression pattern indicates that *OsbZIP60* rather than *OsbZIP39* plays a central role in ER sensing and UPR signaling in rice-developing grains. We next analyzed the impact of HS on the cellularization window in TEJ-5 (**Figure 4D**). Under control conditions, endosperms were partially cellularized at 72 HAF and completely cellularized at 96 HAF. However, HS accelerated the grain development, and grains were cellularization by 72 h. To determine whether the developmental stage influenced TEJ-5-specific induction of UPR genes, we examined the cellularization window for other accessions and found that TEJ-4 is cellularized by 72 h of HS as well (**Supplementary Figure S4**). A similar cellularization pattern in TEJ-4 and TEJ-5 suggests that the induction of UPR genes in TEJ-5 is not due to precocious endosperm cellularization.

In addition to the induction of UPR genes, our analysis yielded a group of iron deficiency-responsive genes that are preferentially induced in TEJ-5 at 72 h of HS (**Figure 4E**). Genes involved in iron homeostasis play a critical role in optimal UPR response, as they regulate *IRE1* clustering propensity and localization (Cohen et al., 2017). Iron also serves as a metal cofactor in the synthesis of many proteins needed to cope with higher respiration and cellular damage under HS. Given the temporal induction pattern of iron and UPR genes, we hypothesize that iron status-associated transcriptional responses precede UPR stress response. Collectively, our morphometric and transcriptome analyses suggest that UPR activation may uniquely contribute to HS tolerance in TEJ-5.

### Group B: grain developmental stage-regulated genes

**Figure 5:**
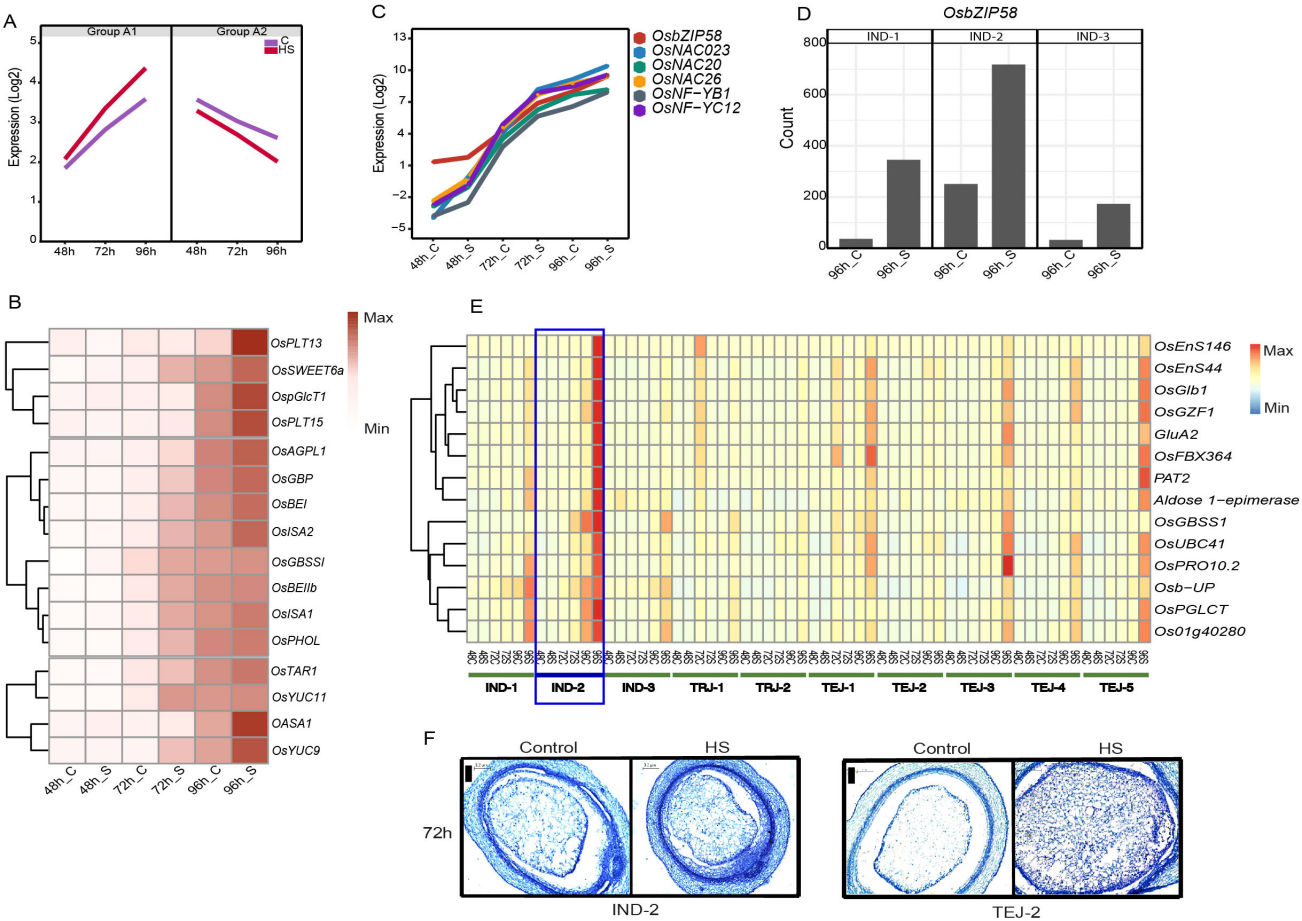
The grain filing mechanism in IND-2 under heat stress (HS). **(A)** Mean expression of genes involved in cell division and early grain filling (group A-1 and group A-2). **(B)** Induction of genes involved in sugar transporter, starch biosynthesis, and phytohormones in IND-2 under HS. **(C)** Expression of starch biosynthesis-related transcription factors induced in IND-2 under HS. **(D)** Expression of *OsbZIP58* at 96 h of HS in IND-2. **(E)** Downstream targets of *OsbZIP58* involved in grain filling in IND-2. **(F)** Histochemical analysis showing the completion of cellularization window of IND-2 at 72 h under control and HS. Scale = 0.2 μm.

We classified genes in clusters 5, 6, 7, 9, and 10 as “early grain-filling regulators”, considering the similar trend in their expression pattern across accessions regardless of the genotypic differences. Transcript abundance of genes in clusters 5, 6, and 7 (group A-1) increased steadily from the coenocytic to cellularization stage, wherein HS further induced their basal expression at 72 h and 96 h (**Figure 5A**). In contrast, the transcript abundance of genes in clusters 9 and 10 (group A-2) declined as the grains progressed from 48 to 96 HAF under control and HS. Group A1 genes were enriched in GO terms such as “nutrient reservoir activity”, “lipid storage”, “protein biosynthesis”, “DNA replication”, and “cell cycle process”. Group A-1 includes 42 HS-responsive sucrose and starch biosynthesis genes and endosperm cellularization markers *SSIIa*, *TPP8*, *ZmEBE-1*, *OSTF1*, *CLSA9*, and an expressed protein, which indicates that A-1 mainly constitutes the sink capacity and grain filling-related genes. In addition to starch biosynthesis genes, A-1 is also populated with storage protein-coding genes, including 14 prolamines, 13 glutelins, and five albumins (**Supplementary Table S6**). A striking difference in the gene expression pattern was observed in IND-2 at 96 h of HS, as several classes of grain-filling genes were more highly induced relative to other accessions. Since the morphometric characterization labels IND-2 as moderately tolerant at the single-grain level, we analyzed whether the distinct expression pattern for grain-filling genes compensated for sgw despite having a significant reduction in grain width under HS. Our analysis showed that monosaccharide transporters (*OspGlcT1*, *OsPLT13*, and *OsPLT15*), SWEET transporters (*OsSWEET6a* and *OsSWEET15*), and a sucrose transporter (*OsSUT1*) are induced in IND-2 under HS, or these genes have relatively high expression under HS in IND-2, suggesting a less disrupted sugar flow to the sink organs (**Figure 5B**). Higher expression of one of these transporters, *OsSUT1*, is observed under an increase in endogenous sugar levels and shows that *OsSUT1* is regulated by endogenous sugar status (Matsukura et al., 2000). Moreover, HS regulated the starch pathway genes (*OsAGPS2a*, *OsAGPL1*, *OsAGPL2*, *OsSSIIa*, *OsSSIIIa*, *OsGBSSI*, and *OsGBP*) in IND-2 in a similar manner. Among other genes involved in various steps in starch biosynthesis, two branching enzymes (*OsBEI* and *OsBEIIb*), two debranching enzyme genes (*OsISA1* and *OsISA2*), and plastidial starch phosphorylase *OsPHOL* showed a similar expression in IND-2 at 96 h of HS. HS also induced phytohormone-related genes that facilitate grain filling, such as YUCCA pathway-mediated IAA biosynthesis genes *OASA1*, *OsTAR1*, *OsYUC9*, and *OsYUC11* in IND-2 at 96 h of HS.

We found that HS activated several TFs involved in starch synthesis in IND-2 (**Figure 5C**). Of these, a central grain-filling pathway mediated by *OsbZIP58* showed significant induction in IND-2 (**Figure 5D**) and, to some extent, in other moderately tolerant or tolerant accessions IND-1, TEJ-3, and TEJ-5 at 96 h of HS. To confirm the regulatory role of *OsbZIP58*, we analyzed its downstream targets (Wang et al., 2013; Kim et al., 2017a). Our analysis showed that 24 downstream genes related to starch and protein synthesis were prominently expressed in IND-2 and other accessions in a pattern matching the *OsbZIP58* upregulation (**Figure 5E**). To address the possibility that higher induction of starch biosynthesis genes is simply not due to more accelerated development under HS, we analyzed the accessions that had similar cellularization stages of IND-2. We confirmed that the cellularization was completed in IND-2 and TEJ-2 by 72 HAF under both control and HS, although *OsbZIP58* targets were induced only in IND-2 (**Figure 5F**). Collectively, these data present evidence for differential pre-programing for grain filling in the transcriptome of developing grains among the accessions under HS at a stage when the endosperm is undergoing cellularization.

We also found that HS misregulated 23 pentatricopeptide repeat (PPR) domain coding genes in group A-1 (**Supplementary Table S6**). Most of the PPR domain coding genes reside in chloroplasts and mitochondria, where their role in RNA editing of the organelle genes influences embryo development and cytoplasmic male sterility (Manna, 2015). As the misregulation of several PPRs is linked to grain developmental defects, we speculate that HS impacts the potential RNA editing function of these 23 PPR genes and could contribute to the early grain developmental defects. Classes of genes associated with cell division and expansion may be determinants of mature grain size, as the number of endosperm cells is related to the grain sink capacity (Lizana et al., 2010). We found cell wall remodeling family genes, including 10 expansins, eight extensins, eight glycosyl hydrolases, 12 glycosyltransferases, two actins, two acetyltransferases, and two methyltransferases, which are likely to influence caryopsis expansion under HS. Group A-1 also has 35 HS-responsive auxin homeostasis genes that may influence the endosperm cell size and density, as auxin promotes cell elongation and differentiation by blocking the POLYCOMB REPRESSIVE COMPLEX 2 (De Lucas et al., 2016; Figueiredo et al., 2016; Paul et al., 2020a). These auxin homeostasis genes also have the potential to regulate grain size by facilitating starch deposition (Zhang et al., 2021). Similarly, group A-1 constitutes five of the 16 rice pumilio family RNA-binding protein genes encoded by the rice genome. Functional implications of pumilio family genes in rice grain maturation have not yet been investigated. However, PUMILIO PROTEIN24, an *Arabidopsis* ortholog of a rice pumilio family gene *LOC_Os03g61560*, has been identified as a positive regulator of grain maturation (Huang et al., 2021b). The function of the *Arabidopsis* ortholog suggests that *LOC_Os03g61560* may also have a role in rice grain maturation under HS.

### Group C: sub-population-preferred genes

Genes in clusters 1, 3, 4, 8, 15, and 16 exhibit sub-population level differences in transcript abundance. We found a set of 47 genes associated with the regulation of redox potential to be preferentially expressed in INDs (Cluster 3) and are likely to influence the divergence of IND rice from japonicas (**Supplementary Table S7**). Differential regulation of these genes in response to HS in one or more accessions could provide insights into the geographical adaptation of rice sub-populations. For instance, allelic variation in one of these redox genes, NADH/NADPH-dependent NO_3_-reductase (NR), *OSNR2*, contributes to the genetic basis of higher nitrate assimilation capacity, nitrogen use efficiency (NUE), enhanced tiller numbers, and grain yield in IND sub-population relative to japonicas (Gao et al., 2019). It is noteworthy that another nitrate reductase gene (*LOC_Os08g36500*) showed TEJ-specific expression and that two NUE-related aminotransferase genes (*LOC_Os03g21960* and *LOC_Os05g15530*) had lower expression in INDs. NUE-related nodulin gene *LOC_Os10g07998* is preferentially induced in IND-2 at 96 h of HS, and expression of a nitrate transporter *OsNRT1.3* is higher in IND-1 and IND-2. Another distinction between INDs and japonicas is attributed to 26 IND-preferred secondary metabolism genes (**Supplementary Table S7**). The end products of these metabolic pathways, such as flavonoids, are accumulated in grain bran and impart various biochemical properties to the grains, or their differential accumulations influence HS tolerance. Since IND-3, the most HS-sensitive accession, naturally has a red pericarp, we examined the activity of anthocyanin biosynthesis pathway genes. We found that HS misregulates anthocyanin structural genes chalcone synthase (*LOC_Os11g32650*), flavanone 3-hydrolase (*LOC_Os04g56700*), flavanone 3′-hydroxylase (*LOC_Os10g17260*), dihydroflavonol reductase (*LOC_Os01g44260*), leucoanthocyanidin reductase (*LOC_Os03g15360*), and anthocyanin regulatory genes such as a bHLH TF regulating proanthocyanidin production (*LOC_Os07g11020*). Either these genes were uniquely expressed in IND-3, or their expression was THE highest in IND-3 compared to other accessions. Therefore, we speculate that a differential accumulation of flavonoids caused by HS may influence the stress sensitivity for IND-3.

Since TEJ and TRJ are less divergent, any differentially regulated genes between these two sub-populations could be potentially involved in adaptation within japonica. In rice domestication, brassinosteroid (BR)-mediated lamina bending to harness light and maximize the yield potential is a superior agronomic trait (Jang and Li, 2017). We found three tandemly arrayed HS-responsive BR INSENSITIVE-1 associated receptor kinase genes in Chr 11 (*LOC_Os11g31530*, *LOC_Os11g31540*, and *LOC_Os11g39370*) along with another BR gene localized to Chr 6 (*LOC_Os06g12120*) to be more strongly transcribed in TRJs (cluster 8). Of these BR genes, *OsBUL1* (*LOC_Os11g31530*) has been shown to positively regulate lamina angle and grain size (Jang and Li, 2017). IND sub-population has a truncated version of a QTL, *OsEBS*, that is responsible for higher biomass and spikelet numbers than japonicas (Dong et al., 2013). Our analysis showed that TRJs exhibit a higher expression level of *OsEBS* compared to TEJs and that truncation causes a reduction in the expression of *OsEBS* in INDs. In addition to fine-tuning of lamina angle, photoperiod sensors contribute to geographical adaptation. Photoperiod regulators *OsMADS50* and *OsPIL1* showed TRJ-preferred expression. Notably, expression of *OsMADS50* was very low in INDs and may have evolved as a japonica-specific photoperiod regulator. The expression of *OsMADS56*, an antagonistic interactor of *OsMADS50* in the long day (LD) flowering regulation (Ryu et al., 2009), is lower in TRJs. Given the natural LD condition in tropical regions, it is possible that these two MADS-box genes play a role in the geographic adaptation of TRJs through flowering time regulation.

Cluster 1 is populated with several photosynthesis-related genes that have lower expression in TRJs (**Supplementary Table S8**). A mutant of one of these photosynthetic genes, *rice Dof daily fluctuations 1*, influences flowering time and grain size (Iwamoto et al., 2009). Similarly, divergence in the promoter sequence of amino acid transporter *OsAAP3* underlies the genetic basis of tiller number and grain yield differences between japonica and INDs (Lu et al., 2018). Such a divergence in expression shows that lower tiller number and grain yield in japonicas are caused by higher expression of *OsAAP3*. In addition to the differential expression analysis between INDs and japonicas, the inclusion of TRJs in our analysis also shows that the expression of *OsAAP3* in TRJs was lower compared to that in TEJs and INDs, hence identifying expression divergence within the japonica sub-population.

## Discussion

In this study, we evaluated the mature grain size and quality of 10 rice accessions under a transient HS and investigated their genetic basis of stress response using transcriptome analysis. A transient HS treatment (36°C/32°C) used in our study was sufficient to classify the accessions into tolerant, moderately tolerant, and sensitive groups. Our analysis showed that 4/5 TEJs (TEJ-2, TEJ-3, TEJ-4, and TEJ-5) showed resilience to HS at the single-grain level, which led us to classify them as tolerant accessions. In contrast, IND-1, IND-2, TRJ-1, TRJ-2, and TEJ-1 showed a significant reduction for one of the evaluated grain size parameters under HS and were classified as moderately tolerant accessions. IND-3 was classified as a sensitive accession, as we detected a significant reduction in all grain size parameters. It is noteworthy that grain width and thickness were most sensitive to HS, wherein 5/10 accessions showed a significant reduction in grain width and thickness under HS (**Figure 1**). However, grain length was not significantly impacted by HS except for IND-3. The phenotypic responses for these traits indicate that the determination of grain width and length is regulated by different pathways with differential heat sensitivity. It is also possible that regulation of grain width to nullify the impact of HS is an adaptive strategy in cereals. Although we found that the nature of transient HS imposed reduced sgw, grain width, and thickness parameters significantly for some accessions but remained unchanged for other accessions, the fertility of marked grains did not change significantly in response to HS. These observations suggest that the intensity and duration of the HS imposition are appropriate for exploring phenotypic variations in HS response at the single-grain level. The imposed stress did not lead to post-fertilization grain abortion, suggesting that the HS during early grain development stages primarily impacts grain size and weight parameters rather than grain number. A high level of sterility could have potentially confounded the single-grain characterization, as lower grain numbers can result in larger grains due to an imbalance between source and sink capacity. Our moderate level of HS treatment mitigated this confounding factor for characterizing single-grain phenotypes. In addition to grain size phenotypic changes, we observed that HS at the early grain developmental stage significantly induced grain chalkiness in some of the accessions (**Figure 1B**; **Supplementary Figure S1B**). In addition to the early developmental stage, the middle stages of grain filling are vital for grain appearance and quality, as this stage is characterized by storage product accumulation (Ishimaru et al., 2009). Therefore, a similar study targeting the middle stages of the grain-filling stage will be useful to identify candidate genes contributing to grain quality.

PCA and DEG analysis observed extensive transcriptome reprogramming proportional to the HS period (**Figure 2**). Only 29.4% of all DEGs (3,582 genes) are common to IND, TEJs, and TRJs. Similarly, 4.2% (511 genes), 14.9% (1,811 genes), and 22.3% (2,710 genes) DEGs are specific to INDs, TEJs, and TRJs, respectively. These estimates highlight the genetic diversity in HS response mechanisms at a sub-population level. We conducted a network analysis to identify unique stress response pathways activated to alleviate the HS impact. Our analysis of grain-expressed genes yielded a cluster (M24) that contains most of the HSPs and 14 TFs, including seven HSFs and ethylene signaling genes (**Figure 3**). Based on the tight co-regulation pattern with HSPs in M24, we suggest that *OsHSFA6*, *OsHSFA2A*, and *OsHSF6* may act upstream of the heat shock response (HSR) mechanism constituted by genes in this cluster. A similar response mechanism in wheat seedlings regulated by *TaHsfA6f* under HS indicates that the HSR observed in our study may not be grain-specific (Xue et al., 2015). It is also noteworthy that we detected the activation of this network only in TEJs (TEJ-1, TEJ-4, and TEJ-5). A TEJ-specific activation of this pathway may be inferred as an HS response mechanism of rice accessions grown in lower temperature conditions in temperate regions.

We detected the activation of UPR in tolerant accession TEJ-5 at 96 h of HS, marked by the induction of several downstream ER-localized chaperonins regulated by *OsbZIP50* (**Figure 4**). Nevertheless, we could not detect a significant change in the expression of the ER sensor *IRE-1* in TEJ-5, which suggests that *IRE-1* expression is regulated in a temporal-dependent manner.

Among the other two ER chaperonins regulators, we found a similar induction of *OsbZIP60*, whereas *OsbZIP39* expression did not change significantly. Therefore, we suggest that rather than *OsbZIP39*, the UPR in TEJ-5 is channeled through *OsbZIP50* and *OsbZIP60*. We found an induction of iron homeostasis genes unique to TEJ-5 at 72 h of HS. As these iron-related genes are activated in response to an iron deficiency status, we speculate that HS triggers iron deficiency at the grain-filling stage. Moreover, the temporal response of these genes at 72 h of HS shows that iron starvation response precedes the UPR. A possible explanation could be attributed to the role of iron in the UPR pathway. For instance, studies on both human and yeast cells showed that iron homeostasis genes have a role in *IRE-1* clustering propensity that enables an optimum UPR activation, and a deficiency of iron leads to a distortion of the *IRE-1* clustering (Cohen et al., 2017). A similar mechanism to alleviate the HS is also attained through ferroptosis, an iron-dependent cell death characterized by the accumulation of lipid reactive oxygen species (ROS) (Distéfano et al., 2021). Accumulation of unfolded proteins in ER under HS causes excessive glutathione (GSH) consumption to reduce the incorrectly formed disulfide bonds. An inhibition in GSH biosynthesis enzymes, together with protein unfolding, leads to ROS accumulation. Regulated cell death (RCD) in reproductive or vascular development has not been characterized previously. Our study suggests that HS may trigger RCD at the grain-filling stage.

Although the expression pattern of several cell cycle and grain-filling genes (clusters 5, 6, and 7) are similar across 10 accessions, a predominant induction of starch and storage protein genes under HS was evident in IND-2 (**Figure 5**). We found that these grain-filling genes in IND-2 under HS were mainly regulated by *OsbZIP58*, which has been previously identified as a master regulator of starch and storage protein genes by binding to their specific *cis*-elements (Kim et al., 2017a). The *OsbZIP58*-mediated grain filling in IND-2 also was supported by the significant induction of its targets, such as *OsEnS-44*, *ONAC024*, and *ONAC026*, under HS. Since sgw of IND-2 showed less sensitivity to HS, we suggest that an effective grain-filling response under HS compensates for reduced grain width. The molecular cause of the high heat sensitivity of IND-3 remains unaddressed. The phenotype is likely induced due to the post-cellularization defects. Particularly, HS sensitivity of IND-3 may be linked to the differential accumulation of anthocyanin content in its pericarp, as our analysis showed the misregulation of anthocyanin structural genes chalcone synthase (*LOC_Os11g32650*), flavanone 3-hydrolase (*LOC_Os04g56700*), flavanone 3′-hydroxylase (*LOC_Os10g17260*), dihydroflavonol reductase (*LOC_Os01g44260*), leucoanthocyanidin reductase (*LOC_Os03g15360*), and bHLH TF (*LOC_Os07g11020*) involved in proanthocyanidin production under HS. Even though our transcriptome profiles did not capture the expression profiles beyond the cellularization stage, we speculate that the misregulation of anthocyanin genes at an early stage under HS inhibits the anthocyanin biosynthesis in IND-3. Consistently, high temperature-dependent repression of anthocyanin biosynthesis has been shown in *Arabidopsis* (Kim et al., 2017b). A depletion in the anthocyanin content under HS may be harmful to accessions with naturally red or brown pericarp, as these metabolites are likely used to scavenge the ROS under high-temperature conditions (Ren et al., 2023). Apart from accession-specific responses, genes preferentially expressed in specific sub-populations or accessions in our study can be targeted for improving agronomic traits under HS. For instance, in addition to *OSNR2* and *OsNRT1.3*, our analysis showed several NUE and redox genes, including *LOC_Os08g36500*, *LOC_Os03g21960*, *LOC_Os05g15530*, and *LOC_Os10g07998*, which may be the basis of observed differences in yield and biomass among sub-populations. A subset of the sub-population-preferential genes also evolved as photoperiod regulators during geographical adaptation. Overall, this analysis has yielded both sub-population level commonalities and individual accession-specific HS responses at the transcriptome level.

In summary, a transient HS at the early grain developmental stage significantly impacts the mature grain size and quality. The 10 rice accessions used in this study showed variation in response to HS, classifying them as tolerant, moderately tolerant, and sensitive accessions. A dissection of the transcriptome revealed that a tolerant accession, TEJ-5, potentially activates UPR to counteract the HS impact. The UPR in TEJ-5 has evidently been facilitated by the iron starvation response, which precedes the UPR signaling cascade. Similarly, a moderately tolerant accession, IND-2, encodes an efficient grain-filling mechanism mediated by *OsbZIP58*. Network analysis of the grain-expressed genes revealed an HSR pathway unique to TEJs, which is likely evolved as the HS response mechanism of accessions grown in temperate regions. However, a severe HS sensitivity of IND-3 is likely caused by the variation in anthocyanin accumulation in the grain pericarp. The genes and pathways discovered in this work can be useful for cataloging HS responses in the rice germplasm and, if validated through genetic analysis, can yield new targets for improving heat resilience in rice.

## Data Availability

The data presented in the study are deposited in the NCBI SRA under BioProject ID PRJNA855943.

## Supplementary material

Supplementary Figure 1 Morphometrics analysis of **(A)** grain length and **(B)** grain RG pixel ratio.

Supplementary Figure 2 Genes showing temperate japonica preferential expression in M24 in the gene network analysis.

Supplementary Figure 3 Expression of *OsbZIP50* in 10 rice accessions.

Supplementary Figure 4 Histochemical analysis of TEJ-4 at 72 and 96 h of HS.
