# Supplementary figures and images for: Phenotypic and transcriptomic responses of diverse rice accessions to transient heat stress during early grain development
Source: Front Plant Sci. 2024 Aug 15;15:1429697. doi: 10.3389/fpls.2024.1429697 (PMC11358087; doi:10.3389/fpls.2024.1429697)

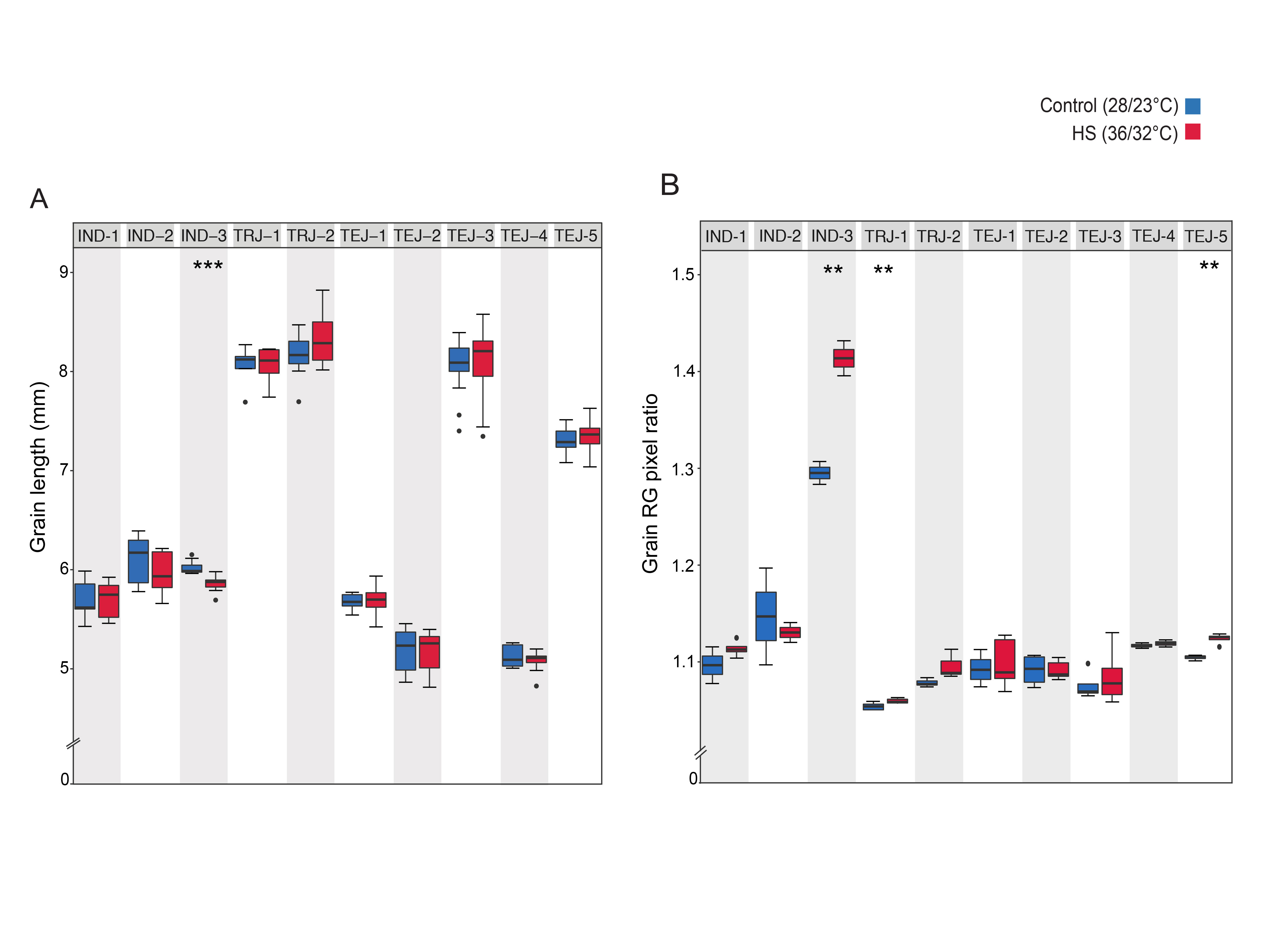

Supplement: Supplementary Figure 1 — Morphometrics analysis of (A) grain length and (B) grain RG pixel ratio. [file Image1.jpeg]

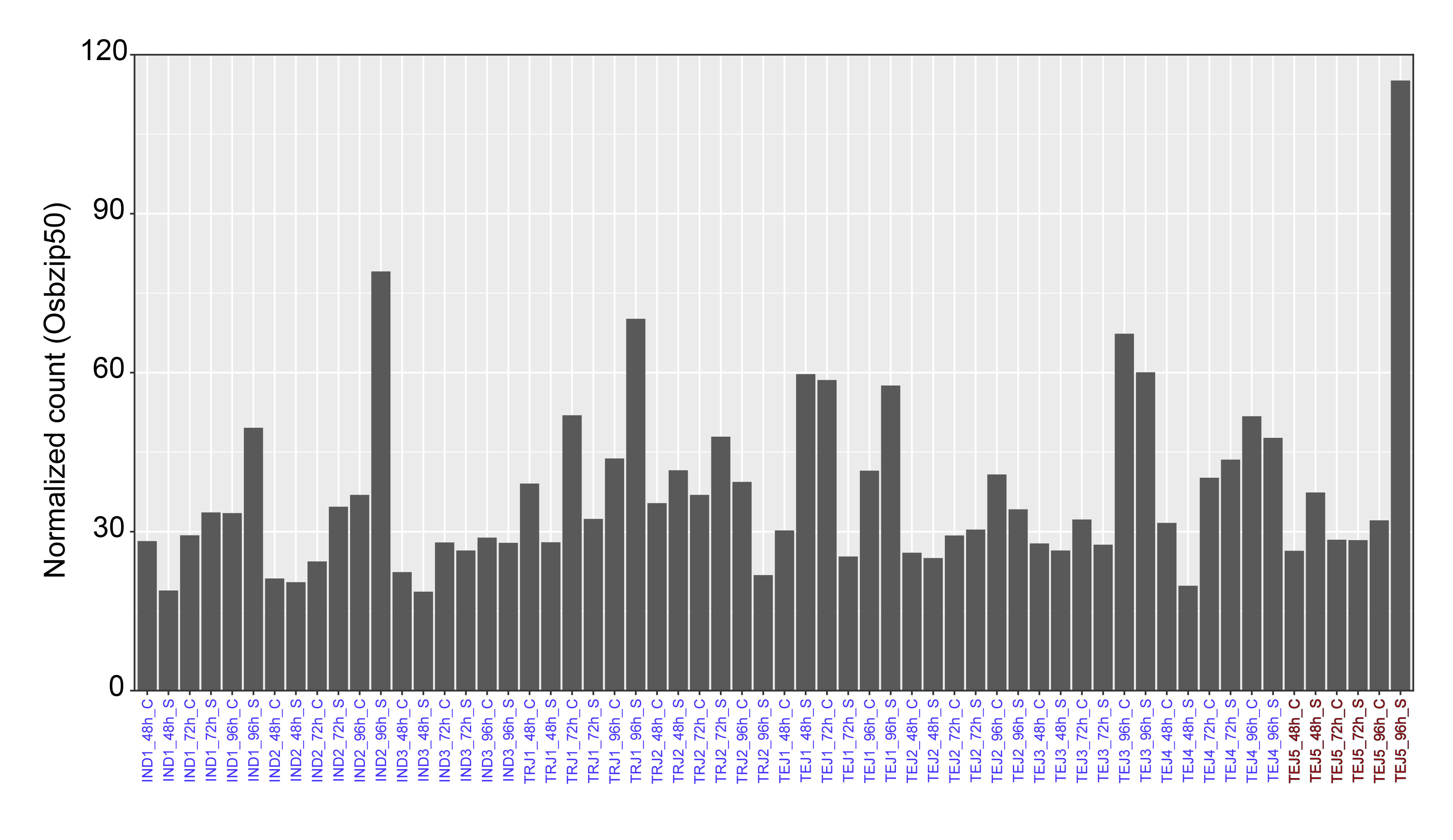

Supplement: Supplementary Figure 3 — Expression of OsbZIP50 in 10 rice accessions. [file Image3.jpeg]

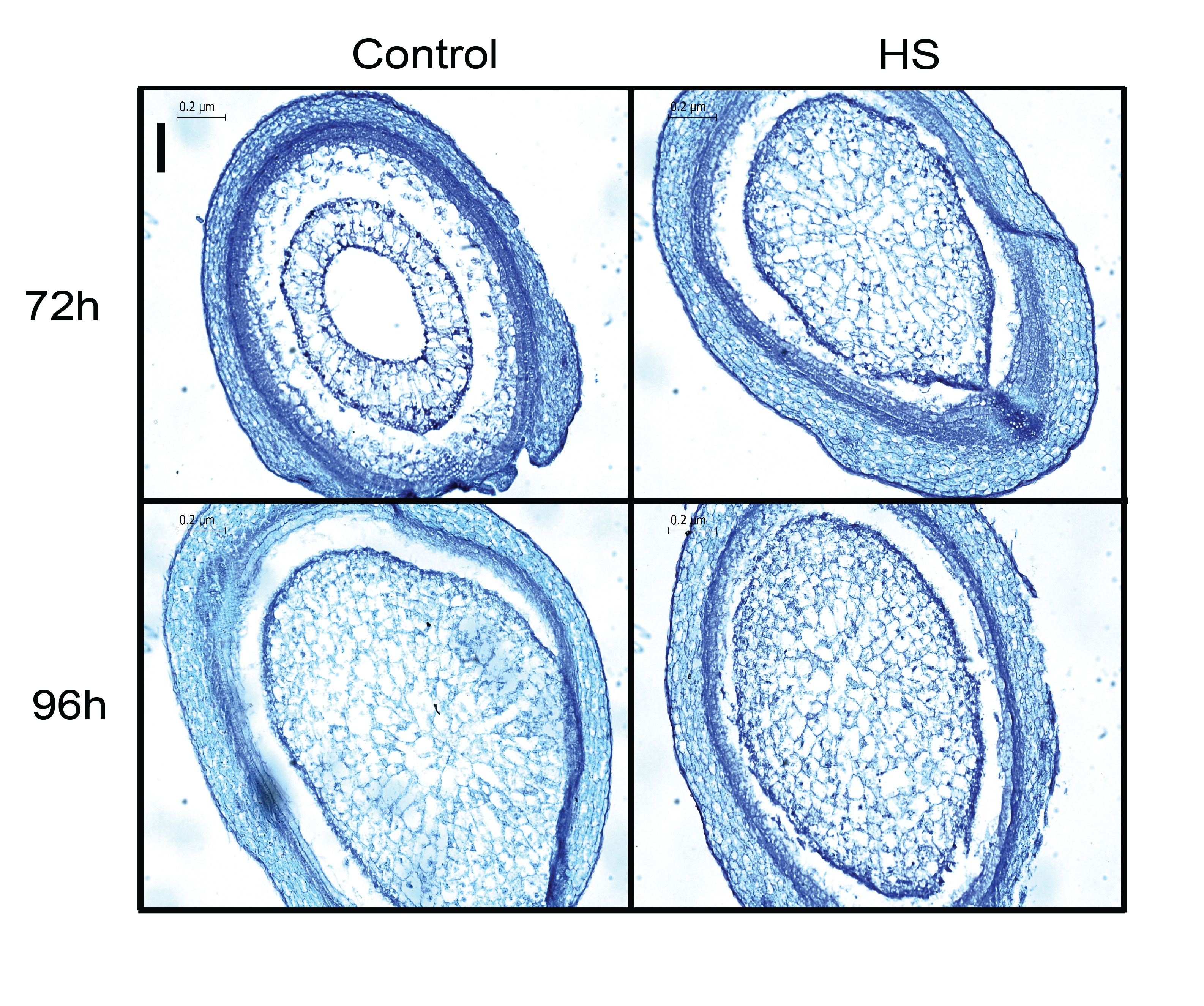

Supplement: Supplementary Figure 4 — Histochemical analysis of TEJ-4 at 72 and 96 h of HS. [file Image4.jpeg]
